# Meta-analysis of experimental factors influencing single-pulse TMS effects on the early visual cortex

**DOI:** 10.3389/fnins.2024.1351399

**Published:** 2024-06-03

**Authors:** Yan Zhang, Bian Song, Xingyue Zhao, Zhenlan Jin, Junjun Zhang, Ling Li

**Affiliations:** MOE Key Lab for Neuroinformation, High-Field Magnetic Resonance Brain Imaging Key Laboratory of Sichuan Province, Center for Psychiatry and Psychology, School of Life Science and Technology, University of Electronic Science and Technology of China, Chengdu, China

**Keywords:** meta-analysis, single-pulse transcranial magnetic stimulation, early visual cortex, experimental factors, visual masking effect

## Abstract

**Background:**

Single-pulse transcranial magnetic stimulation (spTMS) applied to the Early Visual Cortex (EVC) has demonstrated the ability to suppress the perception on visual targets, akin to the effect of visual masking. However, the reported spTMS suppression effects across various studies have displayed inconsistency.

**Objective:**

We aim to test if the heterogeneity of the spTMS effects can be attributable to variations in experimental factors.

**Methods:**

We conducted a meta-analysis using data collected from the PubMed and Web of Science databases spanning from 1995 to March 2024. The meta-analysis encompassed a total of 40 independent experiments drawn from 33 original articles.

**Results:**

The findings unveiled an overall significant spTMS suppression effect on visual perception. Nevertheless, there existed substantial heterogeneity among the experiments. Univariate analysis elucidated that the spTMS effects could be significantly influenced by TMS intensity, visual angle of the stimulus, coil type, and TMS stimulators from different manufacturers. Reliable spTMS suppression effects were observed within the time windows of −80 to 0 ms and 50 to 150 ms. Multivariate linear regression analyses, which included SOA, TMS intensity, visual angle of the stimulus, and coil type, identified SOA as the key factor influencing the spTMS effects. Within the 50 to 150 ms time window, optimal SOAs were identified as 112 ms and 98 ms for objective and subjective performance, respectively. Collectively, multiple experimental factors accounted for 22.9% (*r* = 0.3353) and 39.9% (*r* = 0.3724) of the variance in objective and subjective performance, respectively. Comparing univariate and multivariate analyses, it was evident that experimental factors had different impacts on objective performance and subjective performance.

**Conclusion:**

The present study provided quantitative recommendations for future experiments involving the spTMS effects on visual targets, offering guidance on how to configure experimental factors to achieve the optimal masking effect.

## 1 Introduction

Transcranial magnetic stimulation (TMS) can generate transient magnetic pulses to intervene specific areas of the brain, impacting cognitive functions associated with the intervened brain regions (De Graaf et al., 2014). In 1989, Amassian and colleagues first discovered that a single-pulse TMS (spTMS), applied to the early visual cortex (EVC), asynchronized to the visual target onset, could suppress the perception on the target (Amassian et al., 1989). Since then, extensive studies have investigated the suppression effects of single-pulse TMS on visual perception applied on the EVC (De Graaf et al., 2011a; Koivisto and Silvanto, 2012; Wang et al., 2022). A consistent view suggests that the spTMS works similarly to a visual mask, suppressing the perception on visual targets through a feedforward or feedback projection between the EVC and the higher visual cortex (De Graaf et al., 2014; Hurme et al., 2017). Therefore, spTMS can serve as a novel technique to study the neural mechanism of visual perception.

While spTMS is a reliable masking technique, its specific effects may not be consistently observed across studies. For instance, the spTMS suppression has been observed at different stimulus onset asynchronies (SOA) to the visual target across studies. Previous review articles have proposed a U-shaped suppression with the maximum effect observed between 80 ms and 130 ms (Kammer, 2007), and other dips of suppression occurs around -50 ms, 30 ms, 100 ms and 200 ms (De Graaf et al., 2014). However, the discovery of those dips come from different studies. A dip in one study may not be observed in another. Besides, although most studies demonstrated a suppression effect, some studies have reported enhanced visual perception under spTMS with low magnetic intensity (Abrahamyan et al., 2015), or when spTMS was applied 150 ms or 200 ms before the visual stimulus onset (Mulckhuyse et al., 2011). Some studies revealed an spTMS-induced blindsight (Allen et al., 2014) but some others did not (Koivisto et al., 2010, 2021). Some studies suggested spTMS produced retinotopically specific effects (Jacobs et al., 2014) but other studies also found non-retinotopically effects (Kammer et al., 2005; Salminen-Vaparanta et al., 2014). Retinotopic specific effects pertain to the scenario where visual stimuli are presented either at the location where a spTMS phosphene was generated (Wang et al., 2022) or contralateral to the TMS site (Koivisto et al., 2012). These inconsistencies may due to the fact that different studies adopted different experimental factors. As a consequence, a variety of experimental factors may influence the spTMS effects on visual perception, including SOA, TMS intensity, visual angle of the stimulus, the eccentricity of the stimulus, the placement of TMS coil, the type of TMS coil and TMS stimulators.

In order to systematically investigate which and how the experimental factors influence the spTMS effects, a reasonable approach is to conduct a meta-analysis using data from previous spTMS studies. Compared to the previous review articles (Kammer, 2007; De Graaf et al., 2014), such a meta-analysis can further quantify the impact of experimental factors and investigates potential interactions among them. We aim to provide a comprehensive measurement on the impact of the experimental factors on the spTMS effects in visual perception over EVC.

In the current study, we investigated the spTMS effects applied to EVC, to determine if there is a significant suppression on visual perception and whether this suppression exhibits significant heterogeneity across studies. Then, we examined whether this heterogeneity, i.e., the variations in the spTMS suppression, is correlated with experimental factors, including SOA, TMS Intensity, visual angle of the stimulus, the eccentricity of the stimulus, sample size, the year of publication, coil type and TMS stimulators. Among these factors, SOA is special as previous researches have shown that it does not linearly correlate with the spTMS effects. Therefore, we employed multiple regression analysis to identify the optimal SOA (see method for details) and investigated whether the combination of multiple experimental factors can significantly predict the spTMS suppression effects. These findings could provide a suggestion on the adoptions of experimental factors in the future spTMS studies.

Some of the experimental factors were not included in the analysis, such as the types of visual stimuli and the coil placement (retinotopic or non-retinotopic). Visual stimuli differed across experiments and were task-related. For instance, when the visual stimuli comprised dots, the task might involve judging the number of dots (Hurme et al., 2017), or discerning their motion direction (Laycock et al., 2007). When the task entailed discriminating stimulus direction, the visual stimuli could be bars (Koivisto et al., 2021) or arrows (Jacobs et al., 2012a). As a result, these visual stimuli could not be clearly categorized. Regarding coil placement, although most studies asserted that visual stimuli were located in the receptive field disrupted by TMS stimulation, many did not furnish sufficient evidence. For instance, some studies presented visual stimuli contralateral to the coil placement (Jacobs et al., 2014), while others positioned visual stimuli either to the left or right of fixation with the TMS phosphene appeared at fixation (Perini et al., 2012). Thus, there lacks a definitive standard to distinctly differentiate between retinotopic and non-retinotopic conditions.

In addition, some studies have investigated two types of visual perception: one based on forced-choice tasks, such as discriminating the orientation of arrows or motion directions, detecting whether an object appeared. And another based on subjective ratings of stimulus visibility through self-reported. These two types of tasks allow us to investigate the objective performance under unconscious conditions. It explores whether participants can detect the features of visual targets with an accuracy higher than the chance level, even when they subjectively report not seeing the visual target. If yes, it suggests that spTMS intervention exhibits a phenomenon similar to blindsight. We compared the effect sizes of the studies that measure both the types of tasks to examine whether spTMS can reliably induce blindsight.

## 2 Method

### 2.1 Study selection

**Figure 1:**
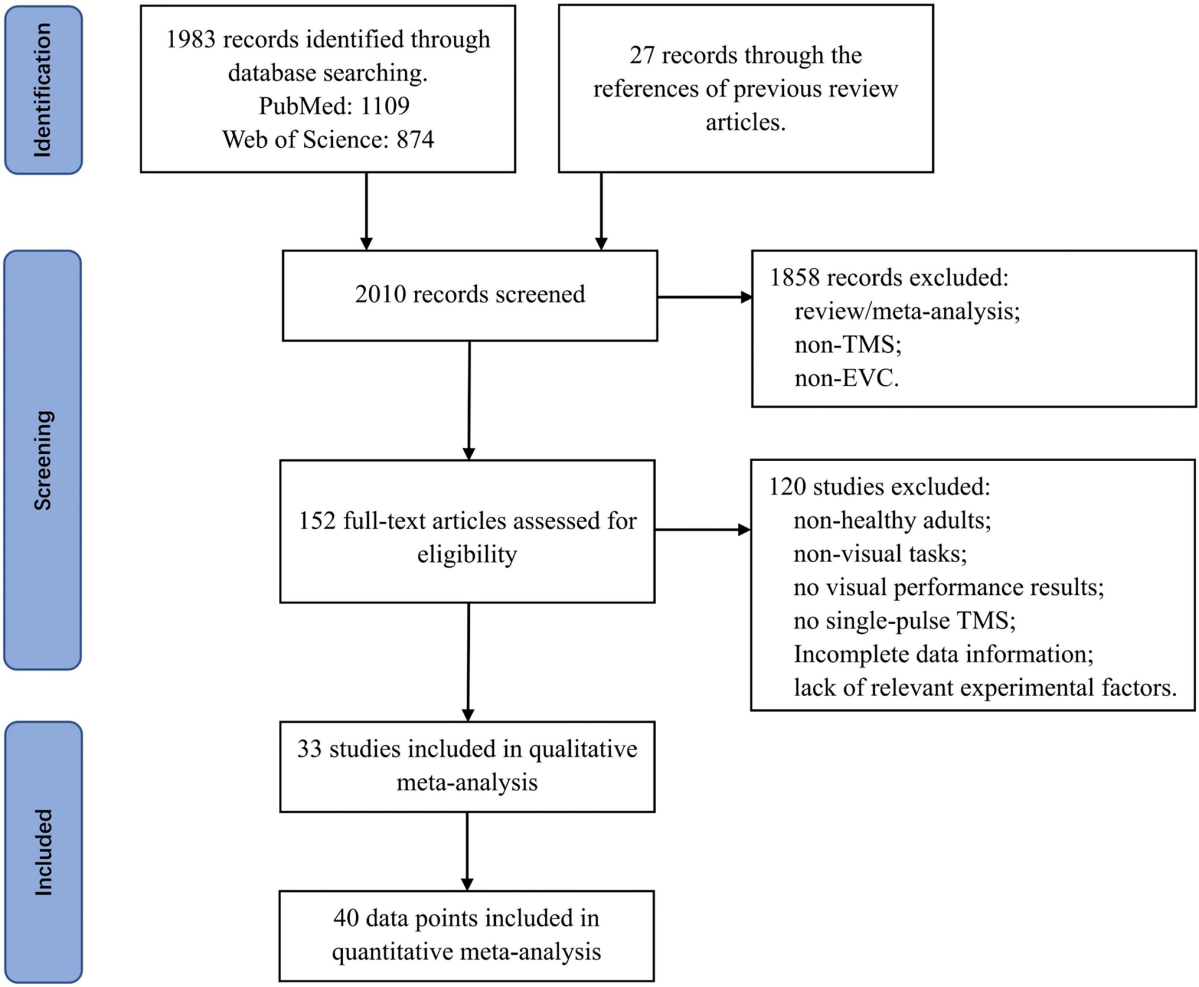
Flowchart depicting the search and selection procedure utilized for the meta-analysis.

To begin with, we conducted a systematic search on PubMed and Web of Science using various combinations of keywords: (“primary visual cortex” or “early visual cortex”) and (“TMS” or “transcranial magnetic stimulation”). We limited our literature selection to articles published from 1995 to March 2024. This was because during the literature search process, we found that articles related to transcranial magnetic stimulation (TMS) before 1995 usually did not use the term “TMS,” but instead used “magnetic coils (MC).” Therefore, we believe that 1995 was an important milestone in the development of TMS technology. Next, we screened titles and abstracts, then conducted a full-text search. In addition, we reviewed the references of previous review articles (Kammer, 2007; Kammer, 2008; De Graaf et al., 2014; Tapia and Beck, 2014) to ensure the inclusion of relevant articles. Please refer to Figure 1 for details of the literature screening process.

### 2.2 Eligibility criteria

1. Original research articles that were published in peer-reviewed English journals.
2. The participants are healthy adults.
3. The inclusion criterion encompassed articles published between 1995 and March 2024.
4. Online TMS was administered to the early visual cortex.
5. The TMS protocols employed was single-pulse stimulation.
6. The experimental results included behavioral performance on the visual task and baseline (no-TMS, vertex or ipsilateral spTMS).
7. All relevant data were either reported in the manuscripts, provided by the authors upon our request, or could be extracted from the graphs presented in the articles.

### 2.3 Data extraction

After conducting the literature search, we identified a total of 33 articles that met the above criteria, encompassing 40 experiments (Table 1). Among these articles, 32 of them provided data on objective visual performance, and 15 of them provided data on the subjective visual performance. The spTMS effects on visual perception were extracted separately for both objective measurements [e.g., reaction time (RT), accuracy (ACC) and d-prime] and subjective measurements [e.g., perceptual awareness scale (PAS)]. In cases where both ACC and RT were reported in a study, we used the ACC results. It was worth noting that different studies used varying chance levels for ACC, which could introduce bias in the effect sizes across studies. To address this issue, we standardized the spTMS effects based on the respective chance levels.

**Table 1:** Articles included.

Study	Measurement	
01 (Hurme et al., 2017)	Objective performance	
02 (Perini et al., 2012)	Objective performance	
03 (Camprodon et al., 2010)	Objective performance	
04 (Romei et al., 2007)	Objective performance	
05 (Abrahamyan et al., 2015)	Objective performance	
06 (Allen et al., 2014)	Objective performance	
07 (Jacobs et al., 2012a)	Objective performance	Subjective performance
08 (Jacobs et al., 2012b)	Objective performance	Subjective performance
09 (Jacobs et al., 2014)	Objective performance	Subjective performance
10 (Railo and Koivisto, 2012)	Objective performance	Subjective performance
11 (Koivisto et al., 2012)	Objective performance	Subjective performance
12 (Laycock et al., 2007)	Objective performance	
13 (Emmanouil et al., 2013)	Objective performance	Subjective performance
14 (De Graaf et al., 2011a)	Objective performance	Subjective performance
15 (De Graaf et al., 2011b)	Objective performance	Subjective performance
16 (De Graaf et al., 2012)	Objective performance	Subjective performance
17 (Ro et al., 2003)	Objective performance	
18 (Beckers and Zeki, 1995)	Objective performance	
19 (Hotson and Anand, 1999)	Objective performance	
20 (Corthout et al., 1999b)	Objective performance	
21 (Hurme et al., 2019)	Objective performance	
22 (Koivisto et al., 2021)	Objective performance	Subjective performance
23 (Wang et al., 2022)	Objective performance	
24 (Hurme et al., 2020)	Objective performance	
25 (Lloyd et al., 2013)	Objective performance	Subjective performance
26 (Cattaneo et al., 2009)	Objective performance	
27 (Koivisto et al., 2010)		Subjective performance
28 (Koivisto and Silvanto, 2012)	Objective performance	
29 (Railo et al., 2014)	Objective performance	Subjective performance
30 (Railo et al., 2012)	Objective performance	
31 (Salminen-Vaparanta et al., 2012)	Objective performance	Subjective performance
32 (Silvanto et al., 2017)	Objective performance	
33 (Olkoniemi et al., 2023)	Objective performance	Subjective performance

In terms of the measurements on the objective visual performance, data were obtained from all 39 experiments, resulting in a total of 930 outcomes from both experimental and control groups. Control groups (including no-TMS, TMS coil positioned at the vertex, or TMS coil positioned over the brain region ipsilateral to the visual stimulus) were utilized as a baseline for assessing the effects of spTMS. The measurements on the subjective visual performance were obtained from 18 experiments, comprising 574 outcomes. An outcome is a set of measurements of the mean score ( ${\bar{X}}_{i}$ ), the standard deviation (*S_i_*), and the sample size (*n_i_*) of the spTMS effects, either from the objective or subjective performance. These experimental factors include: SOA, TMS intensity (the percentage of the maximum stimulator output), visual angle of the stimulus(°), the eccentricity of the stimulus(°) relative to the fixation, sample size, the year of publication, coil type, and TMS stimulators. Coil types were categorized as either circular or figure-of-eight coils, and TMS stimulators were categorized according to their respective manufacturers.

To obtain the above outcomes, for each experiment, we conducted a thorough full-text review to extract relevant information. In cases where the studies did not directly report the required data, we manually extracted the relevant information from the graphs presented in the papers using Plot Digitizer software.^1^ If necessary, we reached out to the authors of the respective articles to obtain any missing or inaccessible data of interest that could not be obtained from the published articles.

### 2.4 Computing the effect sizes

The spTMS effect sizes were calculated using Hedges’ g (Cheung, 2015), which was an improvement on Cohen (1992), based on the above extracted outcomes. Hedges’ g is determined by dividing the standardized mean difference from studies that used two independent groups, i.e., the experimental and the control groups, by the within-groups standard deviation (pooled across groups), and applying a correction factor (J) to address bias in small sample sizes (Michael Borenstein et al., 2009).

The formula for Cohen’s d is


(1) $$d=\frac{\bar{X_{1}}-\bar{X_{2}}}{S_{within}}$$

In which $\bar{X_{1}}$ and $\bar{X_{2}}$ are the sample means of the experimental and control groups, respectively, and $S_{within}$ is the within-groups standard deviation:


(2) $$S_{within}=\sqrt{\frac{\left(n_{1}-1\right)S_{1}^{2}+\left(n_{2}-1\right)S_{2}^{2}}{n_{1}+n_{2}-2}}$$

In which $n_{1}$ and $n_{2}$ are the sample sizes of the two groups, and $S_{1}$ and $S_{2}$ are the standard deviations of the two groups. The correction factor J is calculated as:


(3) $$J=1-\frac{3}{4df-1}$$

In which df is the degrees of freedom used to estimate $S_{within}$ , which equals to $n_{1}+n_{2}-2$ . And the Hedges’ g is calculated as


(4) $$Hedges^{\prime }g=J\times d$$

Finally, 465 and 287 effect sizes were obtained for objective visual performance and subjective visual performance, respectively. In order to control the influence of different types of stimuli on spTMS effects within the same experiment, we averaged the effect sizes of stimuli with varying types while keeping all relevant experimental factors constant. Thus, each of these effect sizes measures the spTMS effects for a specific combination of experimental factors within each experiment, unaffected by the type of stimulus. We referred to the effect sizes as factor-based spTMS effect sizes. In the end, we obtained 316 and 194 factor-based spTMS effect sizes for objective and subjective performance, respectively. Subsequently, for the evaluation of the overall spTMS effects in each experiment, we computed the average effect size for each experiment and denoted it as experiment-based spTMS effect sizes. The experiment-based spTMS effect size is calculated as:


(5) $$\bar{Y}=\frac{1}{m}\left(\overset{m}{\underset{j}{\sum }}Y_{j}\right)$$

In which m represents the number of effect sizes within an experiment, and $Y_{j}$ represents the effect size of the $J_{th}$ effect size.

The variance of the experiment-based spTMS effect size is given by


(6) $$V_{\bar{Y}}={\left(\frac{1}{m}\right)}^{2}\left(\overset{m}{\underset{i=1}{\sum }}V_{i}+\underset{i\neq j}{\sum }\left(r_{ij}\sqrt{V_{i}}\sqrt{V_{j}}\right)\right)$$

In which $V_{i}$ and $V_{j}$ represent the variances of the $i_{th}$ and the $j_{th}$ effect sizes, and $r_{ij}$ is the correlation between the i and j effect sizes. Since all the effect sizes in one experiment were obtained from the same group of participants, they were not independent to each other. Therefore, when calculating the variance, we took into account the correlation among the effect sizes. But due to insufficient prior information, an accurate correlation was unable to estimate. We assumed that the $r_{ij}$ was 0.50 (Michael Borenstein et al., 2009) although the analysis.

### 2.5 Meta-analysis

First, to investigate whether there is a significant spTMS suppression across studies, we conducted a z-test on the experiment-based spTMS effect sizes. Then, we analyzed the variance of the spTMS effects to evaluate the degree of heterogeneity, i.e., the differences across studies. However, the variance included both the true variation in effect sizes and the random error. Thus, the Q statistic was performed to investigate whether there is a heterogeneity across studies, and considering a *p*-value less than 0.01 indicating significant heterogeneity. In addition, the I^2^ statistic was reported to indicate the ratio of true heterogeneity to total observed variation. It demonstrated the signal to noise ratio that revealed the size ratio between true heterogeneity and heterogeneity caused by random errors. I^2^ values of 25, 50, and 75% represent low, moderate, and high heterogeneity, respectively (Higgins et al., 2003). Finally, we used a funnel plot to assess potential bias in the meta-analysis and conducted leave-one-study-out sensitivity analyses to evaluate the stability of the meta-analysis results.

Next, we investigated the impact of experimental factors on the spTMS effects. This analysis was performed on the factor-based spTMS effect sizes. Univariate analysis was performed to investigate the impact of different experimental factors on the factor-based spTMS effect sizes. For factors in continuous forms, including TMS intensity, visual angle of the stimulus, the eccentricity of the stimulus, sample size, and the year of publication, we assessed the linear correlation between the degrees of the spTMS effects and those factors. And for factors in classifiable forms, including coil type and TMS stimulators, we used independent samples t-tests or analysis of variance to analyze whether there were significant differences of the spTMS effects among those factors.

To determine the reliability of the spTMS suppression at various SOAs, we employed a sliding window method with a window length of 20 ms. The window was moved in 10 ms steps, spanning from -100 ms to 300 ms relative to the target onset. By using this approach, we were able to capture fine-grained changes in the spTMS effects over time. The factor-based spTMS effect sizes within each time window from all included experiments were collected. We then conducted two statistical tests: (1) Fisher’s exact test to determine the significance of the spTMS effects compared to zero; and (2) a two-sample t-test, comparing the spTMS effects with the baseline effects. The baseline effects were defined as the collection of factor-based spTMS effects occurring after 250 ms relative to the target onset.

To further our investigation, we sought to determine if there is an optimal SOA for the spTMS effects within different time windows, and whether the combination of multiple experimental factors can reliably predict the spTMS effects. To address these questions, we utilized multiple linear regression analysis. This analysis was performed within the previously identified time window where spTMS was reliably effective. To determine the optimal SOA for the most accurate prediction in each time window, for each iteration, one time point was designated as the supposed optimal SOA. Then the absolute differences between the supposed optimal SOA and all the time points within the time window was defined as the SOA factors. Next, the SOA factors and all the other factors were standardized (z-scored) and subjected to multiple linear regression analysis. To ensure robustness, we employed cross-validation using the leave-one-study-out method. In each iteration of the cross-validation, data from one study were set aside as the test set, while the data from all other studies constituted the training set. The model was then trained based on the training set to derive a multiple linear model, which was subsequently applied to the test data to predict the spTMS effects. This process was repeated for all iterations in the cross-validation, and the correlation coefficient (r value) between the actual and predicted spTMS effects was calculated. Within each time window, an r value was calculated for each time point. The time point with the highest r value was identified as the optimal SOA, as it offered the most accurate prediction for the spTMS effects when considered alongside other experimental factors. By adopting this comprehensive approach, we aimed to identify the optimal SOA and assess the influence of multiple experimental factors on predicting the spTMS effects.

## 3 Results

### 3.1 Meta-analysis of the spTMS effects

**Figure 2:**
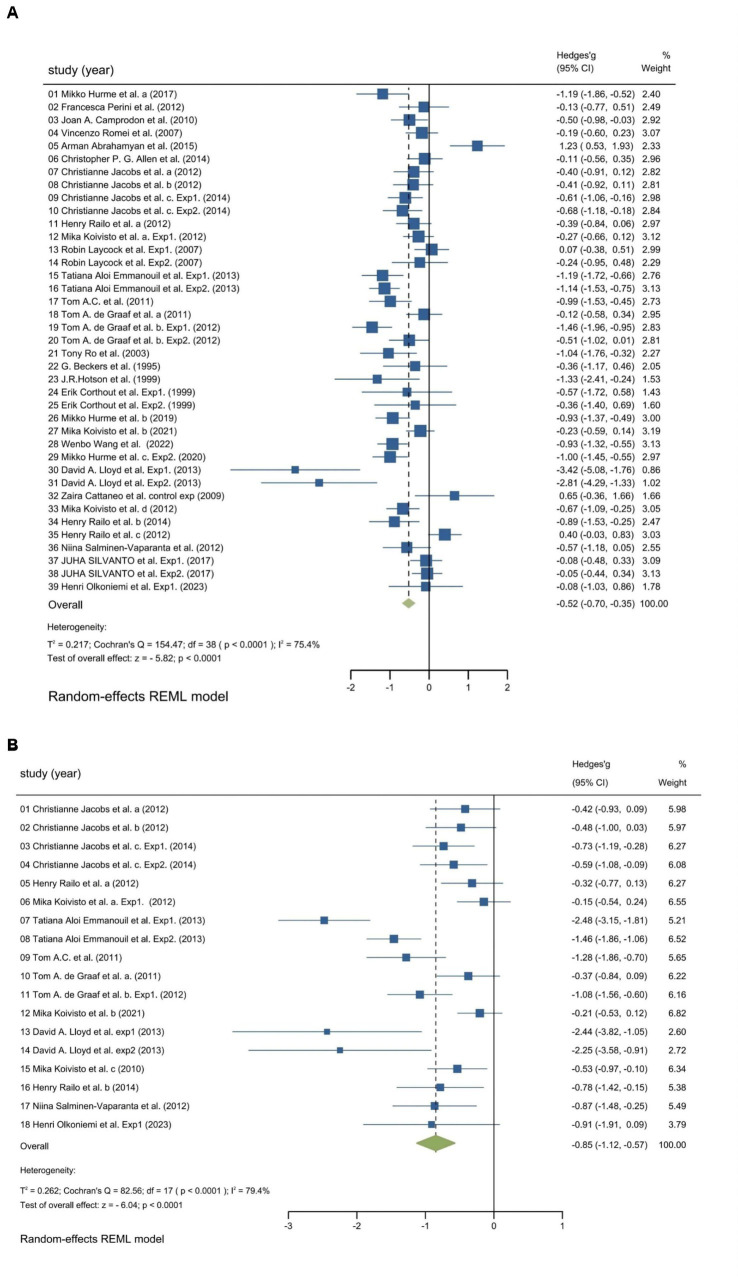
Forest plot. Experiment-based effect sizes for objective performance **(A)** and subjective performance **(B)**. Each square corresponds to an individual experiment’s effect size estimate, with the size of the square reflecting the experiment’s weight. The horizontal line represents the 95% confidence interval (CI). The diamond shape at the bottom represents the overall effect size estimate. The vertical solid line at 0 indicates the null effect. CI, Confidence interval; *I*^2^, Indicators that measure the heterogeneity; Hedges’ g, Effect size indicators.

**Figure 3:**
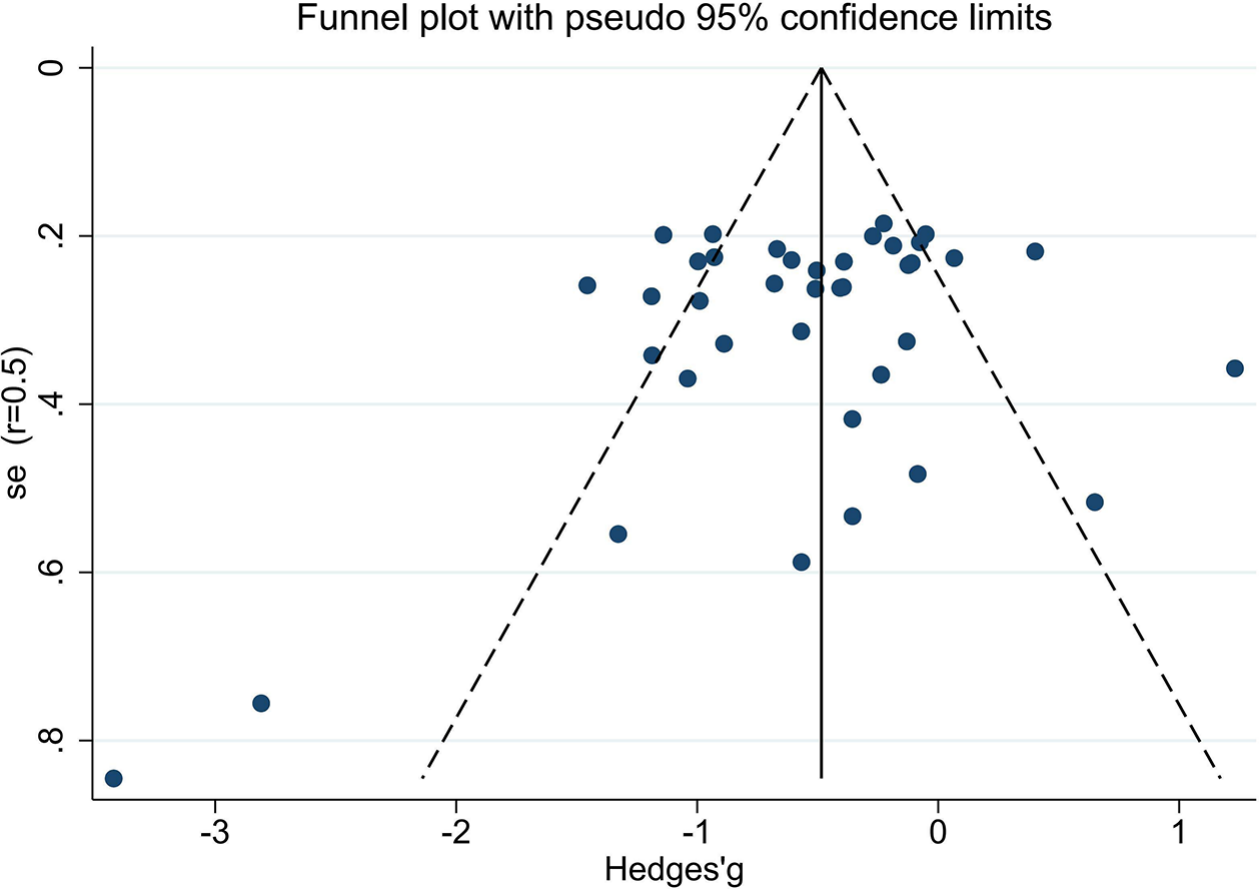
The funnel plot demonstrates the absence of significant bias in the meta-analysis.

**Figure 4:**
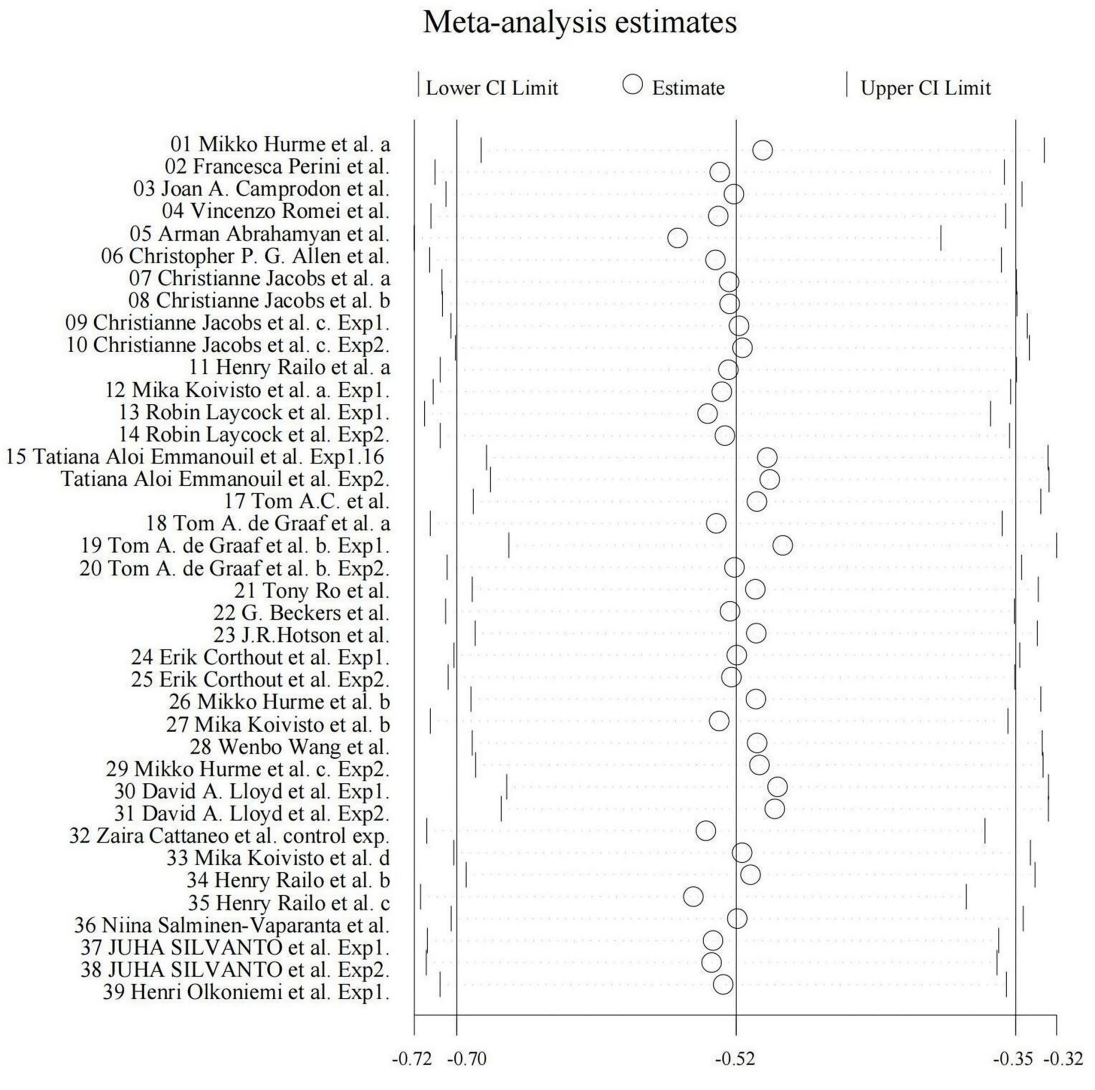
Sensitivity plot. By systematically excluding the data from one experiment at a time, the overall effect size for the remaining experiments (k – 1) can be computed and compared to the overall effect size when considering all experiments (k).

We utilized random-effects models to evaluate the experiment-based spTMS effects for both objective and subjective performance. The results demonstrated that the spTMS had an overall suppression effect on objective performance (overall effect = −0.52, 95% CI: −0.70, −0.35), which was significantly differed from zero (*z* = −5.82, *p* < 0.0001; Figure 2A). There was a significant heterogeneity across experiments (Cochran’s Q = 154.47, *I*^2^ = 75.4%, *p* < 0.0001). The funnel plot analysis (Figure 3) demonstrated a symmetrical distribution of studies, suggesting no significant publication bias among the included studies (*p* = 0.229) as indicated by Egger’s asymmetry test. The sensitivity analysis revealed that excluding individual studies did not result in a significant change in the overall results when compared to the analysis that included all studies (Figure 4). This indicated the stability of the meta-analysis results and supported the reliability of the findings.

The findings regarding subjective performance were similar to those regarding objective performance. There was a significant spTMS suppression on subjective performance (overall effect = −0.85, 95% CI: −1.12, −0.57; *z* = −6.04, *p* < 0.0001), as well as a significant heterogeneity (Cochran’s Q = 82.56, *I*^2^ = 79.4%, *p* < 0.0001), as illustrated in Figure 2B. However, due to the limited number of studies available for subjective performance, bias and sensitivity analysis were not conducted.

### 3.2 Impact of the individual experimental factor on the spTMS effects

**Figure 5:**
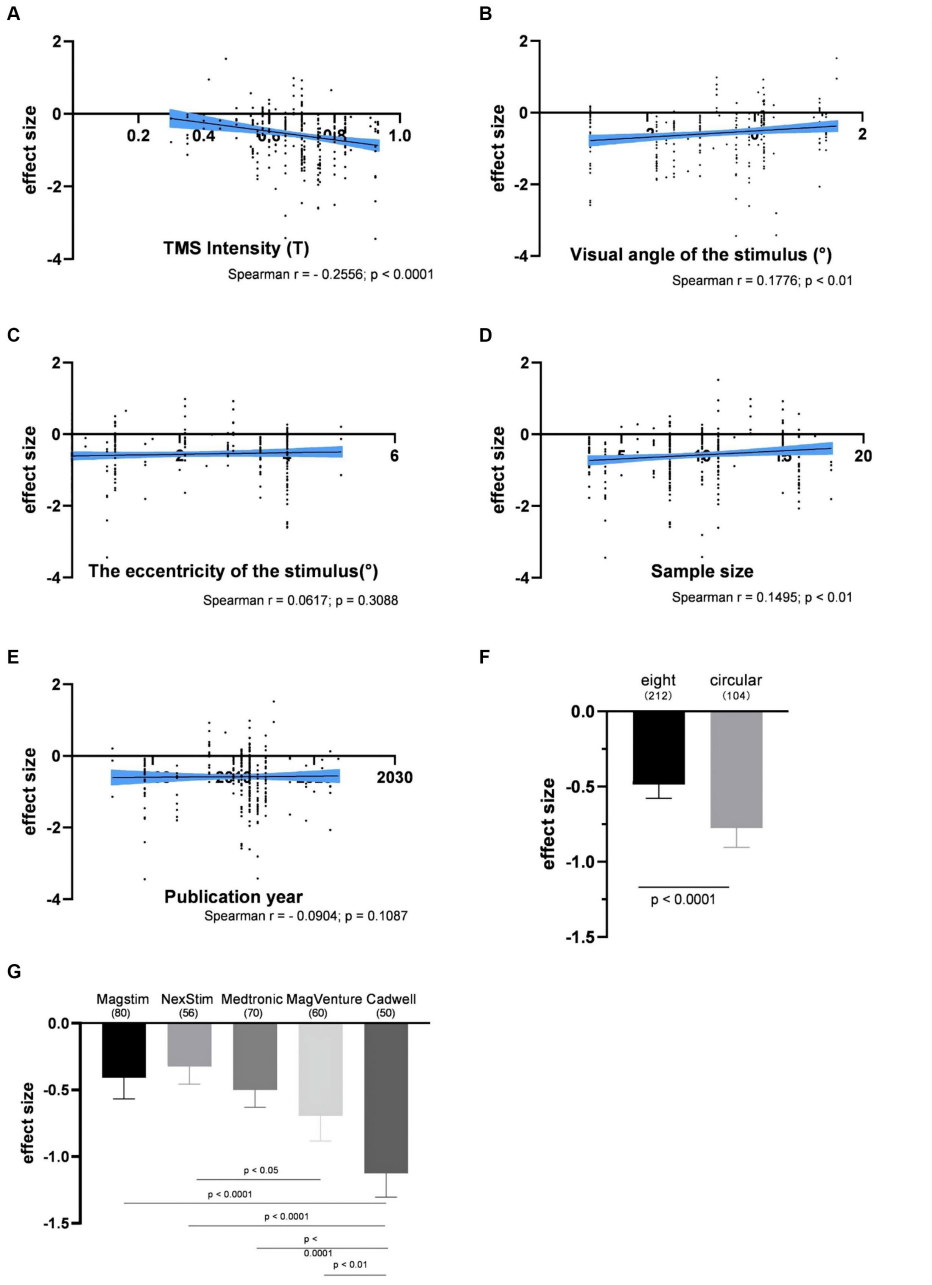
The impacts of experimental factors on the objective performance. **(A)** TMS intensity (*n* = 314); **(B)** Visual angle of the stimulus (*n* = 287); **(C)** The eccentricity of the stimulus (*n* = 274); **(D)** Sample size (*n* = 314); **(E)** The year of publication (*n* = 316); **(F)** Coil type; **(G)** TMS stimulators. The shaded area and error bar represent the 95% confidence interval; All the *p*-values were Bonferroni Corrected.

The impact of the individual experimental factor on the factor-based spTMS effect sizes was investigated. These factors included TMS intensity, visual angle of the stimulus, the eccentricity of the stimulus, sample size, the year of publication, coil type, TMS stimulators, and SOA. Figures 5A–E presented the correlation between the objective performance and the experimental factors in continuous forms. TMS intensity, visual angle of the stimulus and sample size were significantly correlated with the spTMS effects. Increasing TMS intensity exhibited a trend of enhancing the spTMS masking effects (*r* = −0.2556, *p* < 0.0001). Conversely, as visual angle of the stimulus increased, there was a gradual attenuation of the spTMS masking effects (*r* = 0.1776, *p* < 0.01). And as the sample size increased, the spTMS suppression effects weakened (*r* = 0.1495, *p* < 0.01). The eccentricity of the stimulus and the year of publication were not significantly correlated with the spTMS effects. Figures 5F,G showed the impact of the experimental factors in classifiable forms on the objective performance. The results demonstrated that the spTMS effects with the circular coils was found to be more negative compared to the figure-of-eight coils (independent samples *t*-test, *t* = 3.60, *p* < 0.0001). The comparison of the effect sizes was conducted among different TMS stimulators (Magstim, NexStim, Medtronic, MagVenture, Cadwell), considering the known differences in intensity generated by the Medtronic and Magstim systems (Thielscher and Kammer, 2004). The results demonstrated that studies using the Cadwell stimulators exhibited the strongest negative effects, which was significantly different from the effects observed with the other four stimulators.

**Figure 6:**
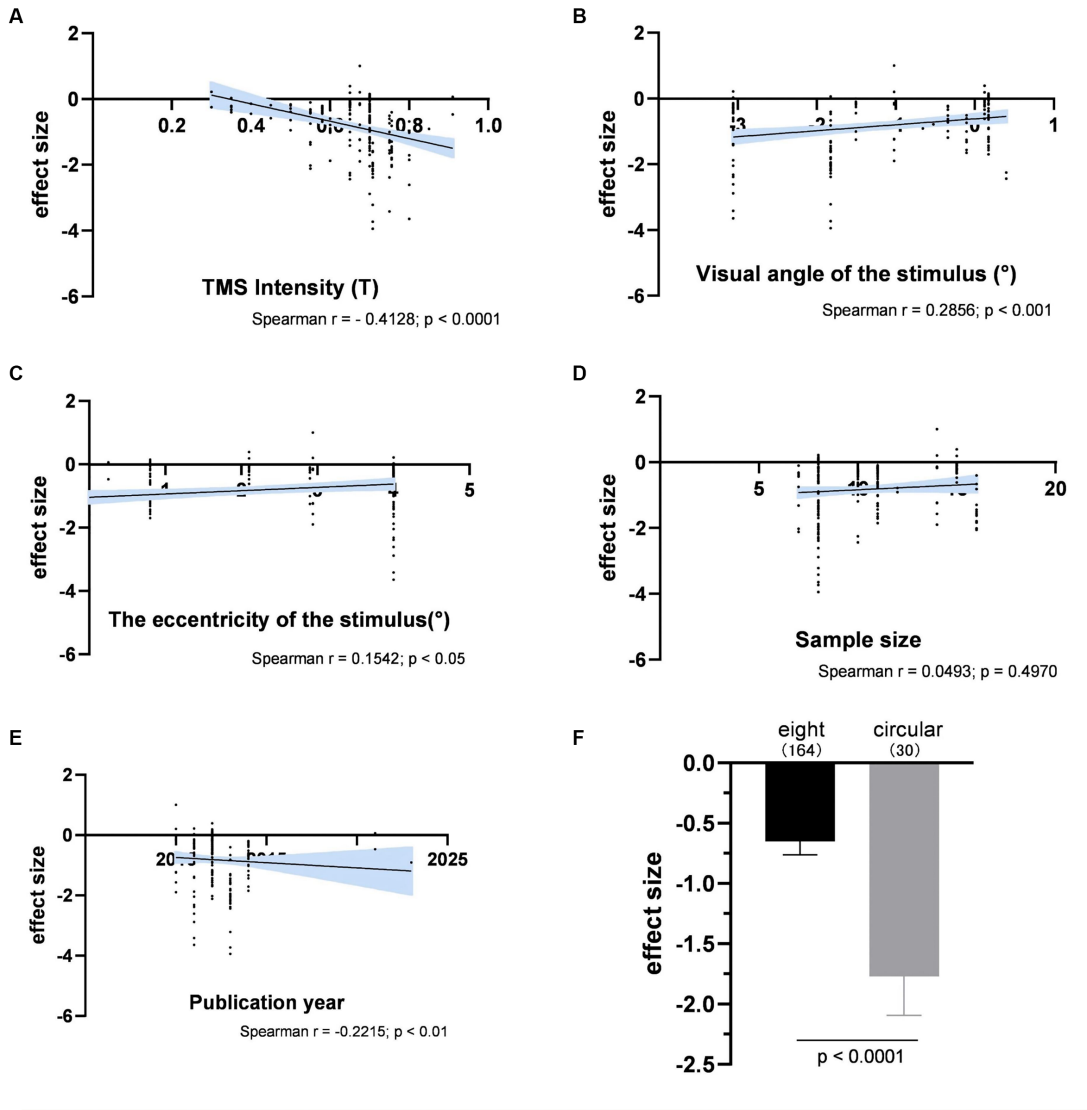
The impacts of experimental factors on subjective performance. **(A)** TMS intensity (*n* = 194); **(B)** Visual angle of the stimulus (*n* = 165); **(C)** The eccentricity of the stimulus (*n* = 163); **(D)** Sample size (*n* = 192); **(E)** The year of publication (*n* = 194); **(F)** Coil type. The shaded area and error bar represent the 95% Cl.

Figure 6 illustrated the impact of various experimental factors on subjective performance. The spTMS effects showed significant correlations with TMS intensity, visual angle of the stimulus, the eccentricity of the stimulus and the publication year. The spTMS masking effects increased with higher TMS intensity (*r* = −0.4128, *p* < 0.0001) and more recent publications (*r* = −0.2215, *p* < 0.01), and decreased with larger visual angle of the stimulus (*r* = 0.2856, *p* < 0.001) and larger the eccentricity of the stimulus (*r* = 0.1542, *p* < 0.05). Due to the uneven sample sizes of experiments employing figure-of-eight and circular coils in the subjective performance (figure-of-eight: 15 experiments with a total of 164 data points; circular: 3 experiments with 30 data points), the Bootstrap method was employed to perform 1,000 iterations with replacement for each of the two samples. In each iteration, 30 data points were randomly drawn from each sample to ensure the equivalence in sample size. The result showed that circular coils produced the stronger spTMS suppression effect (*t* = 170.65; df = 999; *p* < 0.0001) than the figure-of-eight. Furthermore, significant differences were observed in sample sizes across different TMS stimulators, making it inappropriate to analyze the impact of TMS stimulators on the spTMS effects.

**Figure 7:**
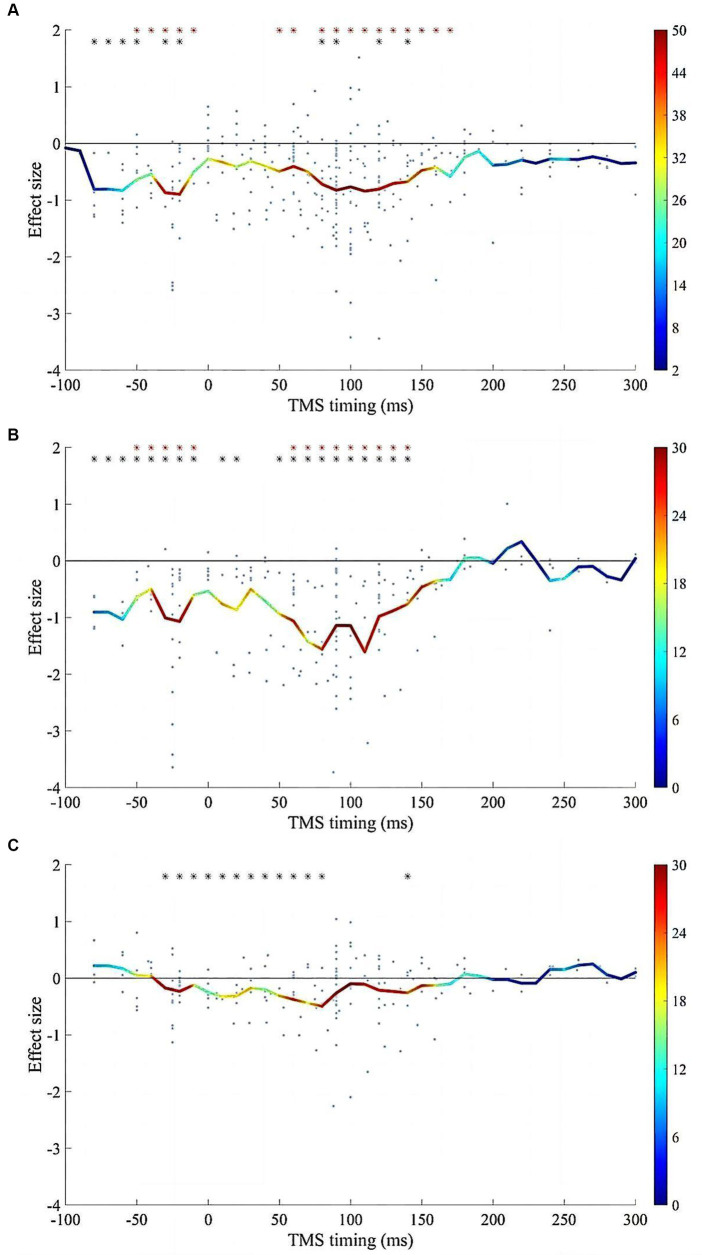
The impacts of SOA on the spTMS effects. **(A)** Objective performance (*n* = 316); **(B)** Subjective performance (*n* = 194); **(C)** The difference between subjective and objective performance (*n* = 184). The red asterisks indicate significant differences in effect sizes when compared to a value of 0. The black asterisks indicate significant differences in effect sizes when compared to the baseline. The color bar represents the sample size within each time window.

Reliable suppression of spTMS effects was observed within two time windows: −80 to 0 ms and 50 to 150 ms, for both objective and subjective performance (Figure 7). Within both time windows, the spTMS suppression was either greater than the null or greater than the baseline spTMS effects. In studies that examined both objective and subjective performance, the effect sizes of the two were subtracted within each sliding time window. However, the fisher’s exact test did not detect any significant difference compared to the null hypothesis, indicating the impact of spTMS on the objective and subjective visual performance when applied to EVC did not show significant differences.

### 3.3 Impact of the multiple experimental factors on the spTMS effects

We conducted multivariate linear regression analyses within −80 to 0 ms, and 50 to 150 ms time windows, to investigated the impact of the multiple experimental factors on the spTMS effects. Experimental factors that were found to significantly impact spTMS effects, such as SOA, TMS intensity, visual angle of stimulus and coil type, were included in the analysis. However, due to variations in sample sizes across coil types in subjective performances, this factor was not included in the subjective multivariate regression model. To address interactions among experimental factors, the following steps were taken: pairwise correlation analyses were initially conducted among the factors. Subsequently, significant interaction terms were integrated into the multiple regression model as control variables. The interactions between SOA and visual angle of the stimulus, as well as between TMS intensity and coil type, were integrated in the objective multivariate regression model. The interactions between SOA and visual angle of the stimulus, and between TMS intensity and visual angle of the stimulus, were integrated in the subjective multivariate regression model.

**Figure 8:**
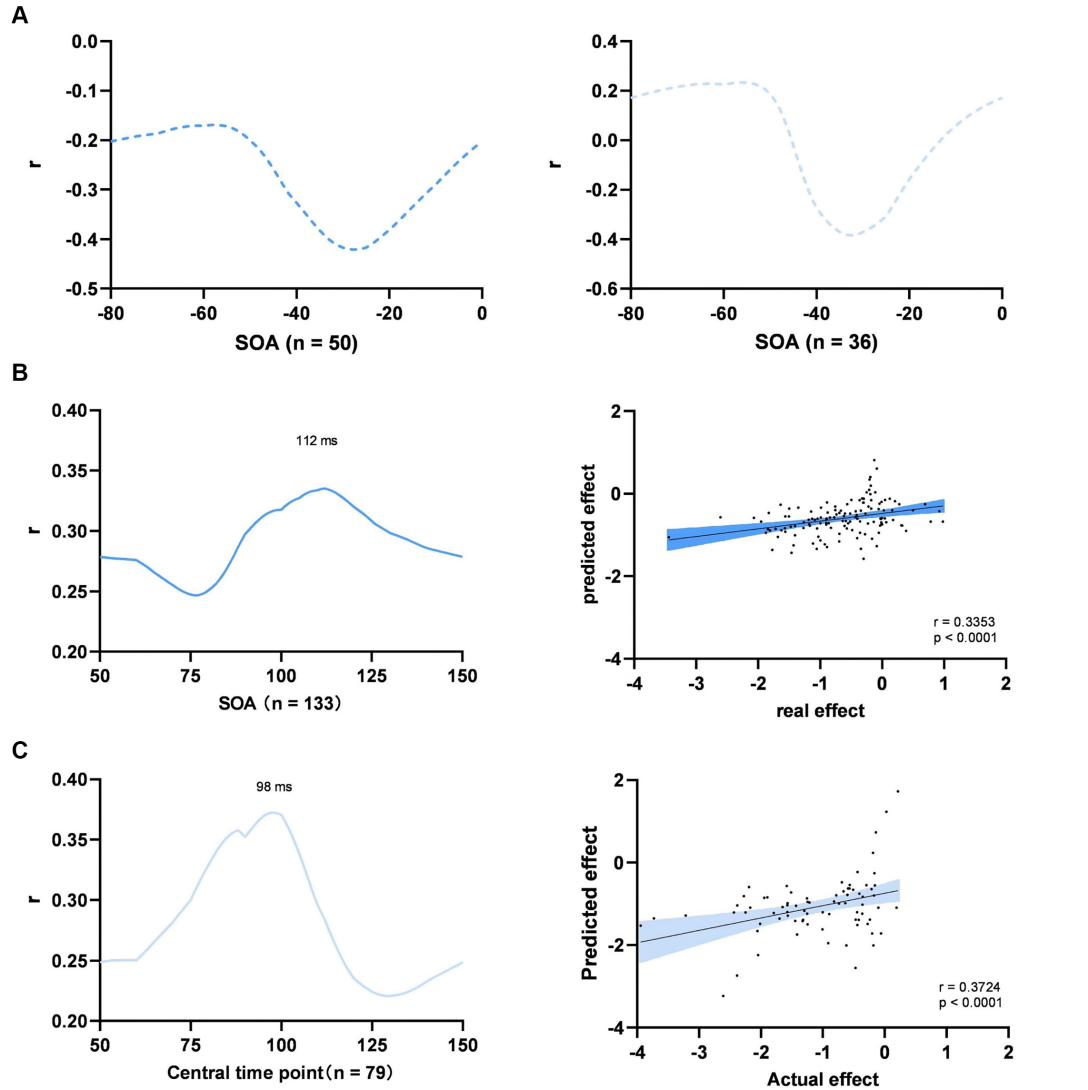
Multiple experimental factors. **(A)** Searching for the optimal SOA of objective performance (left) and subjective performance (right) between −80 to 0 ms; **(B)** Objective performance between 50 to 100 ms; **(C)** Subjective performance between 50 to 100 ms. Left: searching for the optimal SOA; Right: prediction of the model at the optimal SOA; The shaded area represents the 95% Cl.

We identified the optimal SOAs within two distinct time windows: a pre-stimulus window from −80 to 0 ms and a post-stimulus window from 50 to 150 ms. In the pre-stimulus time window, the optimal SOA was not identified, because the multivariate regression model failed to predict the spTMS effects for any time point assumed to be the optimal SOA (Figures 8A). Within the 50 to 150 ms time window, the optimal SOAs for objective performance and subjective performance were found to be 112 ms and 98 ms, respectively (Figures 8B,C). We subsequently converted SOAs into absolute time differences relative to the aforementioned optimal SOAs and incorporated them as variables in the multivariate regression analysis.

In the case of objective performance, the model accounted for 22.9% of the variation in the spTMS effects within the 50 to 150 ms time window (*p* < 0.0001). According to this model, the predicted effect sizes exhibited a significant correlation with the actual effect sizes (Pearson r = 0.3353, *p* < 0.0001). In order to compare the relative impact of each parameter, we conducted multiple regression analyses on all the data, discarding the leave-one-paper-out method. The results demonstrated that the spTMS suppression in objective performance can be significantly predicted by the SOA (*β* = 0.1720, *p* < 0.01), TMS intensity (*β* = −0.5972, *p* < 0.01) and visual angle of the stimulus (*β* = 0.5420, *p* < 0.01). In terms of subjective performance, the model explained 39.9% of the variance (*p* < 0.0001), and the predicted effect sizes were also significantly correlated with the actual effect sizes (Pearson *r* = 0.3724, *p* < 0.0001). The SOA (*β* = 0.3336, *p* < 0.001) and visual angle of the stimulus (*β* = −2.0371, *p* < 0.05) retained significant predictability for the spTMS suppression (Table 2).

**Table 2:** Multiple linear regression.

Predictor	*β*	SE	*t*	*P*	*R*	*R* ^2^	adj.*R*^2^	*F*(P)
50 to 150 ms, objective performance (*n* = 133)								
SOA	0.1720	0.0566	3.0406	0.0029	0.5138	0.2640	0.2290	7.55 (*p* < 0.0001)
tms_intensity	−0.5972	0.2208	−2.7049	0.0078				
visual angle of the stimulus	0.5420	0.1856	2.9201	0.0041				
coil_type	−0.8243	0.4261	−1.9344	0.0553				
SOA × visual angle	−0.4167	0.1807	−2.3052	0.0228				
tms_intensity × coil_type	0.8627	0.5263	1.6391	0.1037				
50 to 150 ms, subjective performance (*n* = 79)								
SOA	0.3336	0.0811	4.1133	p < 0.001	0.6611	0.4370	0.3990	11.3 (*p* < 0.0001)
tms_intensity	0.0852	0.2581	0.3300	0.7424				
visual angle of the stimulus	−2.0371	0.8900	−2.2889	0.0250				
SOA × visual angle	−0.0182	0.2199	−0.0826	0.9344				
tms_intensity × visual angle	2.0537	0.7903	2.5986	0.0113				

## 4 Discussion

The current study conducted a meta-analysis to explore the impact of various experimental factors on spTMS applied over the EVC in visual perception. The study uncovered two key findings. First, in line with previous studies (De Graaf et al., 2012, 2014; Jacobs et al., 2012b; Hurme et al., 2017), spTMS consistently suppresses visual perception, in both objective performance and subjective performance. However, substantial heterogeneity exists across different studies due to varied experimental factors employed. Through univariate analysis, the suppression effect of spTMS was significantly correlated with SOA, TMS intensity, visual angle of the stimulus, coil type and TMS stimulators. The multiple regression model revealed a combination of experimental factors could predict the spTMS effects on objective performance and subjective performance. These findings offer quantitative guidance for configuring experimental factors in future studies to optimize spTMS effects. Second, we found differences in the influences of combined experimental factors on spTMS effects between objective and subjective performance.

### 4.1 Stimulus onset asynchronies

In a previous review article (De Graaf et al., 2014), various SOAs were identified as effective for the spTMS masking. These SOAs included the classical 80-130 ms dip, the pre-stimulus −50-0 ms dip, a ~ 30 ms dip, and a late 200 ms dip. In the current study, results reaffirmed the stability of the classical and pre-stimulus dips across different studies. Furthermore, within the classical dip, spTMS applied at 112 ms was identified as the optimal SOA for objective performance. At this optimal SOA, the correlation between predicted spTMS effects and actual spTMS effects was maximized. It was supposed that the classical dip delineated the timing at which visual information enters and exits the EVC (Amassian et al., 1989). More recent studies have leaned toward the idea that such an optimal timing for spTMS intervention pertains to interrupting a feedback processing of visual information (Kammer, 2008; Hurme et al., 2019).

Within the pre-stimulus time window, significant spTMS effects were also observed between −80 and 0 ms. Previous studies suggested that spTMS may induce a specific state in the occipital cortex unfavorable for subsequent information processing and may disrupt expectations or attentional processes (Laycock et al., 2007). However, unlike the classical dip, the pre-stimulus dip varies across different studies. Jacobs et al. (2012a,b) found that the pre-stimulus dip primarily occurred within −80 to -40 ms. Other studies have subdivided this time window into two distinct dips: dip 0 (~ − 50/~ − 40 ms) and dip X (~ − 10 ms) (De Graaf et al., 2014). Dip 0 exhibited significant suppression effects that were not specific to spatial location after eliminating blinks (De Graaf et al., 2015). Conversely, dip X exhibited specificity based on the TMS site and the position of the visual stimulus (Corthout et al., 2003). However, the optimal SOA within this time window was not identified in the current study. This may indicate that within the pre-stimulus time window, the impact of SOA on spTMS effects is unstable and related to the specific experimental tasks. Nonetheless, it is also plausible that the limited data points within the pre-stimulus time window may also lead to the failure to identify the optimal SOA.

This study did not uncover any significant effects of spTMS in the ~30 ms dip, which was proposed to be caused by the disruption of feedforward processing of visual information. While some previous studies have reported the existence of the 30 ms dip (Corthout et al., 1999a,b; Salminen-Vaparanta et al., 2012), this phenomenon has not been consistently replicated in other studies (Camprodon et al., 2010; Allen et al., 2014; Koivisto et al., 2017; Wang et al., 2022). Several studies have posited that it takes around 50-60 ms for visual information to arrive at the primary visual cortex, casting doubt on the likelihood of the ~30 ms dip occurring (Baseler and Sutter, 1997; Di Russo et al., 2002; Vanni et al., 2004). Additionally, it has been suggested that the color of the visual stimulus influences the efficacy of visual suppression (Emmanouil et al., 2013). Furthermore, Paulus et al. conducted an experiment employing achromatic stimuli and proposed that the ~30 ms dip might be attributed to the earlier involvement of the magnocellular pathway compared to the parvocellular pathway (Paulus et al., 1999). These findings suggest that the~30 ms time window could be stimulus-specific. The late 200 ms dip did not exhibit significant effects across studies either. This may be due to the observation that this dip appears to occur primarily in response to relatively complex stimuli or tasks involving figure-background segregation (Heinen et al., 2005; Camprodon et al., 2010), or in visual search experiments that rely on joint features (Koivisto and Silvanto, 2012). Such a dip cannot be consistently reproduced when using simple or static stimuli (Koivisto et al., 2012; Jacobs et al., 2012a,b).

### 4.2 The interaction of TMS intensity, TMS stimulators and coil type

The spTMS suppression effects became more pronounced as TMS intensity increased, which aligns with previous studies (De Graaf et al., 2011a, 2014; Silvanto et al., 2017). Visual stimuli elicited varying levels of activation in neurons within the visual cortex. Neural populations responsible for processing the visual stimulus exhibited more pronounced activation compared to those not involved in processing it. Such a pattern of neural activities enables specific stimulus recognition. Some studies have suggested that low-intensity spTMS was adequate for activating neurons only relevant to the stimulus, thus enhancing behavioral performance (Silvanto and Cattaneo, 2021). Conversely, high-intensity spTMS activated neural populations that are typically less activated, disrupting the pattern of neural activity for stimulus recognition (Abrahamyan et al., 2015). Thus, as the intensity of TMS increases, the signal-to-noise ratio gradually decreases, and the impact of spTMS on visual performance shifts from facilitation to suppression (Pellegrini et al., 2018; Silvanto and Cattaneo, 2021). However, the maximum stimulator output (MSO) varied across TMS stimulators from different manufacturers (Van Doren et al., 2015). We were unable to obtain the MSO for each TMS stimulator used in the respective studies. Therefore, defining TMS intensity as the percentage of MSO may not have yielded accurate results.

**Figure 9:**
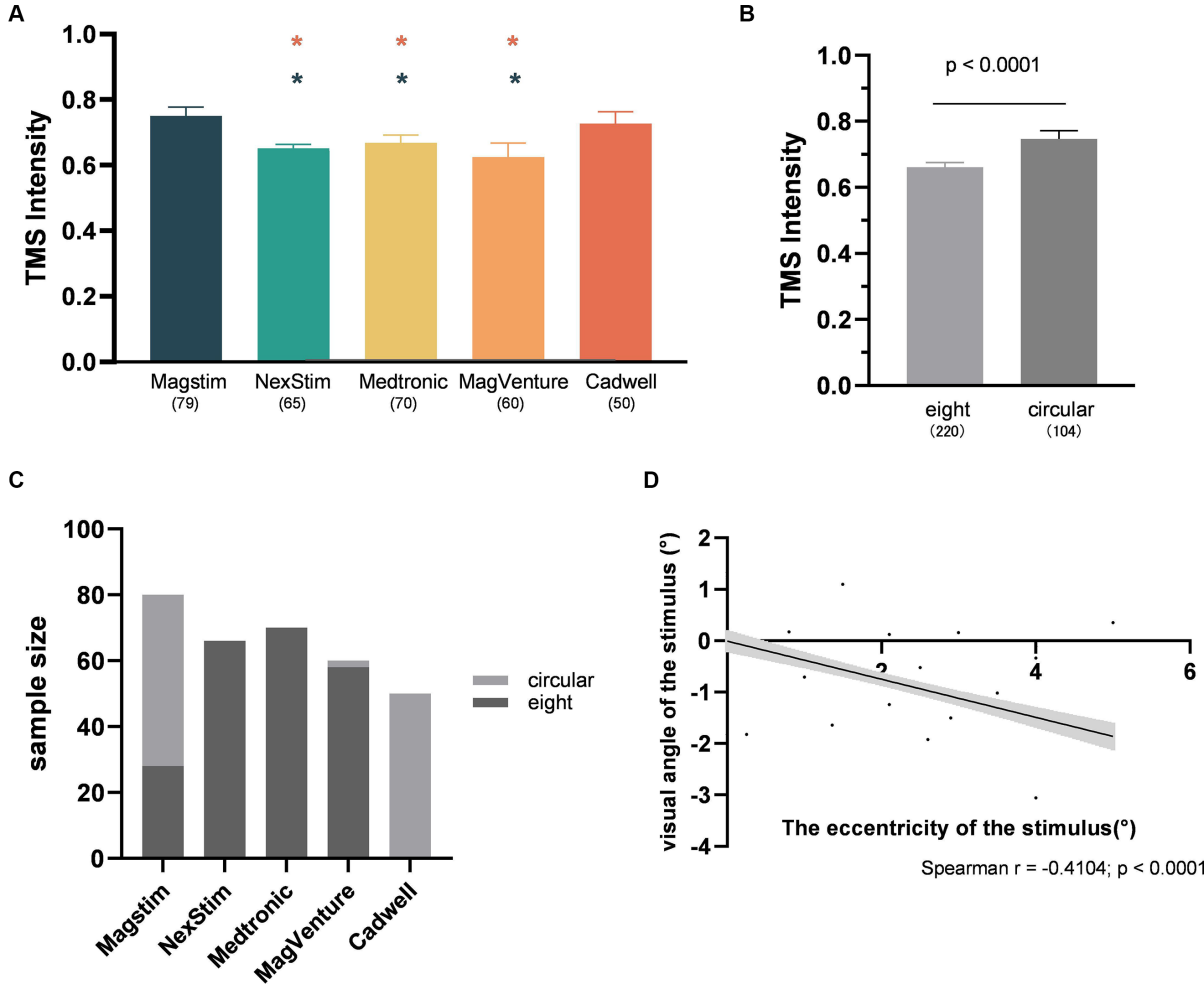
Interaction analysis. **(A)** The differences in TMS intensity among different TMS stimulators (Red star: Significant difference compared to CadWell; Green star: Significant difference compared to Magstim; All the p-values were Bonferroni Corrected.) **(B)** The differences in TMS intensity between coil types; **(C)** The proportion of different coil types used in different TMS stimulators; **(D)** The correlation between the visual angle and eccentricity of the stimulus (*n* = 284). The shaded area and error bar represent the 95% Cl.

This study revealed that the CadWell stimulator exhibited the strongest spTMS suppression effects, significantly outperforming Magstim, NexStim, Medtronic, and MagVenture stimulators (Figure 5G). Three factors account for this variance. Firstly, there are variations in magnetic field intensity among different stimulators. Research has demonstrated that Medtronic stimulators produced a stronger field strength compared to Magstim stimulators (Thielscher and Kammer, 2004; Lang et al., 2006). Secondly, TMS stimulators from different manufacturers may possess distinct characteristics in terms of the generated pulses (Kammer et al., 2001). Studies have shown that spTMS effects on neurons can be influenced by factors such as the waveform of the pulses delivered by the stimulator (Brasil-Neto et al., 1992), as well as the geometric shape and orientation of the induced electric field (Amassian et al., 1992). Thirdly, studies employing different stimulators also shown significant differences in the application of TMS intensity. In experiments using the Cadwell stimulator, the selected TMS intensity, expressed as percentage of MSO, was significantly higher than that used NexStim, Medtronic, and MagVenture stimulators (Figure 9A).

It is noteworthy that the spTMS effects are also influenced by the type of the coil used. Circular coils produced stronger suppression effects compared to figure-of-eight coils (Figures 5F, 6F). Figure-of-eight coils deliver maximum TMS intensity at the intersection of their two circular coils, concentrating the stimulation deeply in a localized area. In contrast, circular coils yielded peak TMS intensity in a central ring, affecting a broader area (Jalinous, 1991; Deng, et al., 2013). However, the choice of coil type was also associated with the selection of TMS intensities and stimulators. Researchers tended to use stronger TMS intensities when employing circular coils (Figure 9B). Studies utilizing CadWell stimulators exclusively employed circular coils (Figure 9C).

Thus, in the studies included in the current meta-analysis, because of the associations among TMS intensity, TMS stimulator and coil type, the spTMS effects cannot be attributed solely to any one of them. The interactions among the three factors need to be considered in the multivariate linear regression analysis.

### 4.3 The visual angle and eccentricity of the stimulus

The suppression effect of spTMS increased as visual angle of stimulus decreased (Figures 5B, 6B). Previous studies rarely directly investigated the impact of visual angle of stimulus on the spTMS effects. This intriguing phenomenon may also be attributed to spTMS preferentially activating neural populations with lower levels of activation, which reduces the signal-to-noise ratio (Silvanto and Muggleton, 2008). As visual angle of the stimulus decreased, the corresponding activated brain regions also decreased in size (Stacchi and Caldara, 2022). Consequently, when applying spTMS, it may disrupt the entire brain regions associated with a small stimulus but only affect part of the brain regions corresponding to a relatively large stimulus. As a result, spTMS appears to be more effective at masking a small stimulus.

The current study did not uncover a significant correlation between the eccentricity of the stimulus and the spTMS effects in objective performance. However, a noticeable association was observed between the eccentricity and the visual angle of the stimulus (Figure 9D). Directly interpreting the relationship between eccentricity and the visual angle of the stimulus was challenging, as the selection of both factors depends on specific experimental tasks. Future research should explore the influence of eccentricity on the spTMS effects, as well as its relationship with the visual angle of the stimulus and experimental tasks.

### 4.4 The differences between objective and subjective performance

The phenomenon where objective performance under conditions of invisibility remained higher than the chance level was termed as spTMS-induced blindsight (Railo and Koivisto, 2012; Koenig and Ro, 2019). In this study, no differences were observed between objective and subjective performance of spTMS effects (Figure 7C). However, it cannot be simply interpreted as the absence of spTMS-induced blindsight, because whether spTMS can induce blindsight depends on various factors, such as stimulus features (size, color, static or dynamic), the precision of TMS site, and task complexity. Railo and Hurme proposed TMS may induce blindsight of stimulus presence or location. However, the current study could not investigate this due to the limited relevant data points (n = 16) on such tasks (Railo and Hurme, 2021). Additionally, there has been debate on the approach to subjective consciousness assessment, with some arguing that individuals’ reported unconsciousness may not genuinely equate to a state of unconsciousness. In instances where graded scales were employed to assess subjective consciousness, the spTMS-induced blindsight was not observed (Koivisto et al., 2021).

### 4.5 The impact of multiple experimental factors

The current study employed a multiple linear regression model incorporating various factors. For objective performance, the interaction between SOA and visual angle of the stimulus, as well as the interaction between TMS intensity and coil type were controlled in the model. Results revealed that SOA, TMS intensity and visual angle of the stimulus collectively predicted the spTMS effects. In terms of subjective performance, the interaction between SOA and visual angle of the stimulus, as well as the interaction between TMS intensity and visual angle of the stimulus were controlled in the model. The multivariate regression model could significantly predict the spTMS effects. Among the experimental factors, SOA and visual angle of the stimulus reached significant levels. Furthermore, different experimental factors exhibited varying degrees of importance in predicting the spTMS effects, with SOA emerging as the most significant predictor for the spTMS suppression effects.

Coil type and TMS intensity were not significant in objective and subjective performance, respectively. This findings suggest that, in the context of the spTMS impacts, the individual predictive power of TMS intensity and coil type alone is insufficient. The significant role of these factors in univariate analysis is likely attributed to the interactions among these factors. Additionally, the significance of experimental factors differed between subjective and objective performance models. This result reveals differences in the influences of combined experimental factors on spTMS effects between objective and subjective performance.

### 4.6 Limitations

There are some limitations to the current study. Firstly, insufficient data posed a primary challenge when examining spTMS effects. The uneven distribution of sample sizes across different levels for several factors (such as sample size, the year of publication, coil type and TMS stimulators) potentially hindered a comprehensive understanding of spTMS effects. Particularly, low sample sizes for certain factors (such as TMS stimulators in subjective performance) may have led to null findings or even made statistical analysis infeasible. Additionally, key factors such as expectation levels and attention levels were not reported in many studies included in this meta-analysis, yet they were crucial for understanding the impact of TMS effects (Battistoni et al., 2017). Furthermore, the TMS baseline was essential for interpreting spTMS effects, but the use of different measurement methods (including no-TMS, TMS coil positioned at the vertex, or TMS coil positioned over the brain region ipsilateral to the visual stimulus) in various studies made analysis challenging.

## Data availability statement

The original contributions presented in the study are included in the article/Supplementary material, further inquiries can be directed to the corresponding authors.

## Author contributions

YZ: Data curation, Formal analysis, Investigation, Software, Visualization, Writing – original draft. BS: Formal analysis, Investigation, Visualization, Writing – original draft. XZ: Formal analysis, Validation, Writing – original draft. ZJ: Writing – review & editing. JZ: Conceptualization, Funding acquisition, Software, Supervision, Writing – original draft, Writing – review & editing. LL: Conceptualization, Supervision, Writing – review & editing.

## Footnotes

^1^ http://plotdigitizer.com
